# Covid-19 vaccine, apartheid and xenophobia: no need to panic!

**DOI:** 10.4314/ahs.v21i4.1

**Published:** 2021-12

**Authors:** James Tumwine

Welcome to this December issue of *African Health Sciences* in our 21^st^ year of publication. We bring you papers largely on infectious disease which range from Covid-19 to HIV. Interesting, because Covid-19 has reached a watershed, with the announcement of a new variant (Omicron), that has exposed the discriminatory world that had demonstrated vaccine apartheid, and now infectious disease xenophobia. Ironically both situations have some link with South Africa. The word *apartheid* was conceived and implemented in racial South Africa, where black people were treated like they were non-human.^1^ When liberation and independence came, the non-South Africans from neighboring countries that had supported the liberation struggle suffered in a wave of xenophobia.^2^,^3^

And come Covid-19! What happens? The high-income countries buy and hoard vaccines ostensibly to protect their citizens. And when the new Covid-19 variant is detected in southern African countries, what do the high-income countries do? They go into panic mode^4^ and introduce draconian measures to exclude travelers from those countries. Many of those countries have described the action as xenophobia. Unfortunately, Covid-19 and climate change have taught us that this small fragile planet is a small village. When people in one part of the world sneeze all of us are in potential danger. We can pretend that hoarding vaccines or locking out people whose skin color is different from ours will protect us, but this is a fallacy!

What we need is global unity, solidarity and practice universal *ubuntu* - a South African word that means “humanity”. It is also called the “I am because we are” humaneness that puts people first regardless of their color or creed. Unless we practice *ubuntu*, we are very unlikely to control this Covid-19 pandemic!
